# Aberrant Activation of the WNT/β-Catenin Signaling Pathway in Lupus Nephritis

**DOI:** 10.1371/journal.pone.0084852

**Published:** 2014-01-21

**Authors:** Xiao-dong Wang, Xin-fang Huang, Qing-ran Yan, Chun-de Bao

**Affiliations:** Department of Rheumatology, Ren Ji Hospital, School of Medicine, Shanghai Jiao Tong University Shanghai Institute of Rheumatology, Shanghai, PR China; University of Alabama at Birmingham, United States of America

## Abstract

**Objective:**

The canonical WNT pathway has been implicated as playing important roles in the pathogenesis of a variety of kidney diseases. Recently, WNT pathway activity was reported to be elevated in the renal tissue of a lupus mouse model. This study aimed to evaluate the potential role of the WNT pathway in the pathogenesis of human lupus nephritis.

**Methods:**

The expression of β-catenin was evaluated in renal biopsy specimens from lupus nephritis patients and control kidney tissues by immunohistochemistry and western blotting. Real-time polymerase chain reaction (RT-PCR) was used to detect RNA expression of β-catenin, Dkk-1 and Axin2. Plasma concentrations of Dkk-1 were measured by ELISA.

**Results:**

Immunohistochemistry and western blotting revealed increased expression of β-catenin in the kidneys of patients with lupus nephritis compared with control kidney tissues (*p*<0.05), accompanied by an increase in mRNA expression of β-catenin (*p*<0.01) and axin2 (*p*<0.05).

β-catenin was significantly greater in LN patients without renal interstitial fibrosis compared with those with renal interstitial fibrosis (*p*<0.01) at the mRNA expression level; the increase in β-catenin mRNA positively correlated with the creatinine clearance rate (Ccr) and negatively correlated with chronicity indices of renal tissue injury. Greater plasma Dkk-1 concentrations were found in LN patients compared with controls (*p*<0.05). Plasma Dkk-1 concentrations also correlated negatively with anti-dsDNA antibody levels and positively with serum C3 levels.

**Conclusions:**

The canonical WNT/β-catenin signaling pathway was activated in lupus nephritis patients, accompanied by an increase in plasma levels of Dkk-1. Altered WNT/β-catenin signaling was related to the pathogenesis of lupus nephritis and might play a role in renal fibrosis.

## Introduction

Systemic lupus erythematosus (SLE) is an autoimmune disease characterized by the formation of various autoantibodies and multiorgan involvement. The kidneys are the most commonly affected organs, and renal involvement is a major cause of death in lupus patients [1]. The molecular mechanisms of the initiation and progression of lupus nephritis(LN) remain largely unknown.

The WNT signaling pathway is best known for its role in embryogenesis and cancer [2]. The WNT pathway is activated by the binding of the WNT ligand to a seven-pass transmembrane Frizzled receptor and its coreceptor, which prevents the degradation of β-catenin, mediated by glycogen synthase kinase 3β (GSK-3β). β-catenin then accumulates in the cytosol and translocates to the nucleus, where it associates with lymphoid enhancer factor and T-cell factor (LEF-1/TCF) to form a transcription complex, leading to the activation of the genes involved in cell proliferation and differentiation [3].β-catenin, the central component in the canonical WNT pathway, serves as a molecular switch for the WNT pathway. The WNT signaling pathway is tightly regulated by several antagonists, including the secreted Frizzled-related proteins, WNT inhibitory factor, Cerberus and the Dickkopf protein family. Dickkopf-1(Dkk-1) is a downstream of gene of WNT signaling pathway and will be activated by WNT signaling pathway which was reported as a specific inhibitor of WNT/β-catenin signaling [4].

Increased WNT signaling induces the expression of matrix metalloproteinases (MMPs) [5], which may be of importance in extracellular matrix remodeling and the loss of membrane integrity that occurs in lupus nephritis [6]. Recently, Tveita and Rekvig [7] reported increased canonical WNT pathway activity in the kidneys during the development of nephritis in a lupus mouse model (NZB × NZW F1 mice), paralleled by an increase in renal and serum levels of the WNT inhibitor Dkk-1, which suggested that WNT signaling might play an important role during the development of nephritis in the lupus mouse model. However, there was no report of increased activity of this pathway in human lupus nephritis. In the current study, we aimed to explore the potential role of the WNT pathway in the pathogenesis of human lupus nephritis.

## Materials and Methods

### Subjects and controls

This study was approved by the Review Board for Renji Hospital in Shanghai, the Republic of China. Written informed consent was obtained from all study participants. Nighty-seven consecutive patients with lupus nephritis who had been referred to Shanghai Renji Hospital (Shanghai Jiaotong University, School of Medicine) during 2010-2011 for renal biopsy, 34 control specimens, and 40 healthy donors were recruited for this study (Table 1). All lupus patients fulfilled the revised American College of Rheumatology (ACR) classification criteria for SLE [8], respectively. Disease activity of the SLE patients at the time of the study was assessed using the SLE Disease Activity Index (SLEDAI) [9]. Tissues for the comparative control renal samples for each patient were obtained from normal renal tissues 2–5 cm away from the patient's renal malignancy during the same hospital visit.

**Table 1:** Demographic, clinical, and laboratory characteristics of the LN patients.

	Class III	Class IV	Class V	Class V+III	Class V+IV	All
Number (%)	7	18	28	18	26	97
Gender:Male	0	2	1	1	1	5
Female	7	16	27	16	25	92
Age	40.9±11.8	30.0±12.0	30.8±11.7	32.4±11.22	33.9±11.9	32.5±11.8
SLEDAI score	8.86±4.6	9.78±2.8	6.81±4.38	8.11±4.5	8.38±3.4	8.19±4.0
24 h-proteinuria(g/d)*	2.48±3.46	4.69±3.19	2.50±2.49	2.00±2.21	5.01±3.58	3.53±3.2
Ccr(ml/min)*	125.6±54.6	90.8±48.7	132.3±34.7	123.6±35.4	108.1±43.0	116.0±43.3
Dosage of corticosteroid(median mg/d)	60	57.5	35	40	55	50
Albumin*	33.1±3.8	25.7±5.2	31.8±6.4	33.5±6.29	26.7±6.2	29.6±6.7
C3*(mg/ml)	0.54±0.32	0.38±0.13	0.70±0.29	0.62±0.21	0.45±0.20	0.54±0.25
ESR	26.2±12.0	41.0±33.0	33.3±26.8	37.4±26.2	40.2±25.8	37.0±26.9
Disease duration (median months)	94	17.5	19	49	71	31
AI*	4.29±1.5	7.83±2.2	1.61±0.7	3.72±1.7	7.00±2.2	4.79±3.0
CI*	1.86±1.6	3.11±2.3	1.43±1.2	2.33±1.4	3.27±2.1	2.43±1.9

:*p*<0.05.

ESR: erythrocyte sedimentation rate.

C3:serum complement component 3.

### Renal histology and Immunohistochemistry Staining

All patients underwent an ultrasound-guided renal needle biopsy. The renal tissues obtained by biopsy were fixed in 10% neutral buffered formalin, gradually dehydrated, and embedded in paraffin. Sections were stained with hematoxylin-eosin and periodic acid-Schiff reagent using standard protocols. Biopsy specimens were classified using the International Society of Nephrology/Renal Pathology Society (ISN/RPS) 2003 classification of LN [10]. Paraffin-embedded kidney sections (4 µm) were dewaxed in xylene and rehydrated in graded ethanol solutions. Antigen retrieval was enhanced by microwaving the slides in 0.01 M citrate buffer (pH = 6). Sections were incubated with primary anti-β-catenin antibody (Millipore, USA, dilution 1∶300) overnight at 4°C. The sections were then incubated with a secondary peroxidase-conjugated antibody (EnVision™ Detection Kit, Gene Tech, China) at room temperature for 30 minutes. Antibody binding was visualized with 3,3′-diaminobenzidine tetrahydrochloride (DAB) before brief counterstaining with hematoxylin. As a negative control, the primary antibody was replaced by PBS containing 1% bovine serum albumin. The degree of immunohistochemical (IHC) staining was recorded using a semiquantitative and subjective grading system. We evaluated the expression of β-catenin by an independent pathologist, who was blinded to the clinical data. The results were expressed as the staining intensity (0–3) multiplied by the percentage of cells that were positive for β-catenin staining.

### Activity and chronicity indices of renal tissue injury

Renal tissue injury was evaluated using activity and chronicity indices as previously reported by Austin and colleagues [11]. The activity indices was the sum of the scores (on a scale of 1 to 3) for endocapillary proliferation, karyorrhexis, fibrinoid necrosis (with the score for fibrinoid necrosis multiplied by 2), cellular crescents (with the score multiplied by 2), hyaline deposits, leukocyte exudation, and interstitial inflammation. The score on the chronicity indices of renal tissue injury (CI) was the sum of the scores (on a scale of 1 to 3) for glomerular sclerosis, fibrous crescents, tubular atrophy, and interstitial fibrosis.

### Western Blotting

One portion of the fresh renal tissue was snap-frozen in liquid nitrogen immediately and stored at −80°C before use. Proteins of frozen tissues were obtained with RIPA(Thermo Scientific). Proteins were then subjected to sodium dodecyl sulfate–polyacrylamide gel electrophoresis, blotted with the monoclonal rabbit anti-human β-catenin antibodies (Epitomics, USA), and detected with Luminol/Enhancer Solution (Pierce, Rockford, IL). Tubulin antibodies and conjugated secondary antibodies were obtained from cell signaling technology (USA). Relative protein levels were quantified using ImageJ software, version 2.1.4.7 (NIH, USA)

### RNA extraction and complementary DNA (cDNA) synthesis

One portion of the fresh renal tissue was snap-frozen in liquid nitrogen immediately and stored at −80°C before use. Total RNA in the frozen tissues was extracted using TRIzol (Invitrogen, Carlsbad, CA, USA) following the manufacturer's protocol. The quantity and quality of RNA was measured using a NanoDrop™ 1000 spectrophotometer (NanoDrop Technology, Wilmington, DE, USA). Sixty-four samples with 260/280 nm ratios above 1.8 were reverse transcribed into cDNAs using a Reverse Transcription System (cDNA Reverse Transcription Kit, Takara) with random primers.

### Real-time polymerase chain reaction

Quantitative PCR was performed using SYBR green dye for the detection of β-catenin and Dkk-1 mRNA expression in an ABI Prism 7900HT Sequence Detection System (Applied Biosystems). RPL13A was used as an endogenous control. The sequences of the primers were as follows: Dkk-1, forward 5′-CGCAGGCGTGCAAATCT-3′, reverse 5′-TGACGCATGCAGCGTTTT-3′; β-catenin, forward 5′-GGCCTCTGATAAAGGCTACTGTTG-3′, reverse 5′-ACGCAAAGGTGCATGATTTG-3′; Axin2, forward 5′- CGGGAGCCACACCCTTCT-3′, reverse 5′- TGGACACCTGCCAGTTTCTTT -3′, and RPL13A, forward 5′-CCTGGAGGAGAAGAGGAAAGAGA-3′, reverse 5′-TTGAGGACCTCTGTGTATTTGTCAA-3′.

Relative expression levels were calculated using the -ΔΔCt method. Normalization was accomplished by comparing the relative expression of each sample with that of the control sample.

### Enzyme-Linked Immunosorbent Assay (ELISA)

Plasma Dkk-1 levels from SLE patients and healthy donors were measured by enzyme-linked immunosorbent assay using a commercially available test kit (R&D, Minneapolis, MN, USA) according to the manufacturer's protocol.

### Statistical Analysis

Statistical analysis was carried out using GraphPad Prism Software, version 5.0. The results are presented as the mean±SD. The Student's t-test and one-way analysis of variance (ANOVA) were used for determining differences between groups. For heterogeneity of variance, differences were tested with the Mann-Whitney test for the comparison of two groups. Correlations were determined by two-tailed Pearson correlation analysis. Correlation coefficient was applied as the index of measuring correlation, *P* values under 0.05 were considered statistically significant.

## Results

### Demographic, clinical, and laboratory characteristics of the LN patients

The distribution of the ISN/RPS classification of the 97 patients was as follows: 7 were class III, 18 were class IV, 28 were class V, 18 were class V+III, and 26 were class V+IV (Table 1).

Althought some result of statistical analysis was no significant,there was a trend than class IV and V+IV patients was likely to had greater SLEDAI scores, 24-hour proteinuria, and lower levels of serum complement component 3(C3) and albumin. Consistent with the clinical presentation of the disease, the class IV patients also had more disease activity.

### Increased expression ofβ-catenin in the kidneys of LN patients

**Figure 1:**
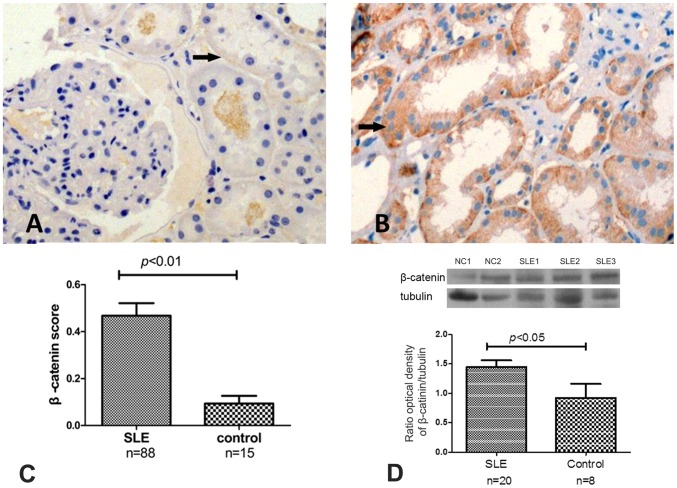
The protein levels of β-catenin in LN and control kidney tissues. The IHC results of β-catenin in LN and control kidney tissues(A and B). The IHC results showed that the staining of β-catenin was significantly stronger in lupus nephritis samples compared with controls (the levels of β-catenin were 0.467±0.500 vs. 0.093±0.129, respectively, *p*<0.01.) (C). Quantification of western blotting revealed significantly increased β-catenin in 20 renal biopsy specimens compared with 8 control renal specimens (D) (1.45±0.11 vs. 0.77±0.21, *p*<0.05).

β-catenin was found to be expressed in the tubular compartments of the glomerulus glomeruli (Figure 1 A, B). The expression of β-catenin was significantly greater in the renal biopsy specimens in 88 LN patients compared with 15 control renal specimens (0.467±0.500 vs. 0.093±0.129, *p* <0.01, Figure 1C). Quantitative analysis of western blotting showed significantly increased β-catenin in 20 renal biopsy specimens compared with 8 control renal specimens (fig. 1D) (1.45±0.11 vs. 0.77±0.21). But we didn’t find that the expression of β-catenin at protein level has correlation with SLEDAI, CI, creatinine clearance rate (CCr) and C3 level.

**Figure 2:**
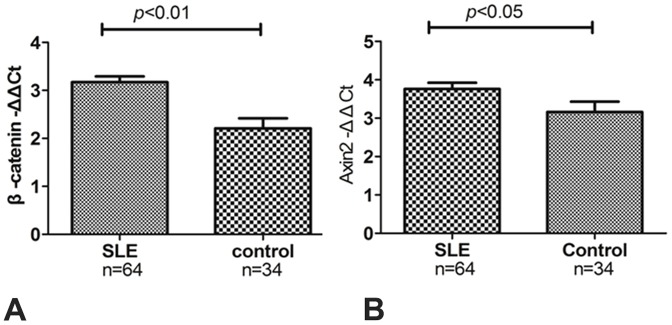
The mRNA expression of β-catenin and Axin2 in LN and control kidney tissues. The mRNA expression of β-catenin in LN patients was significantly greater compared with controls (-ΔΔCt = 3.171±0.122 vs. 2.208±0.201, respectively, *p*<0.01) (A). mRNA expression of Axin2 in LN patients was significantly greater compared with controls (-ΔΔCt  = 3.762±0.160 vs. 3.160±0.271, *p*<0.05,) (B).

Real-time PCR analysis of mRNA isolated from kidney homogenates was performed,which revealed a significant increase in the expression of β-catenin in 64 LN patients compared with 34 controls (-ΔΔCt = 3.171±0.122 vs. 2.208±0.201, *p*<0.01) (Figure 2A). Moreover,Axin2 which as a TCF/LEF-responsive gene was also increased in mRNA expression(-ΔΔCt  = 3.762±0.160 vs. 3.160±0.271, *p* = 0.046, Figure 2B).

### Correlation of mRNA expression of β-catenin with clinical and laboratory characteristics

**Figure 3:**
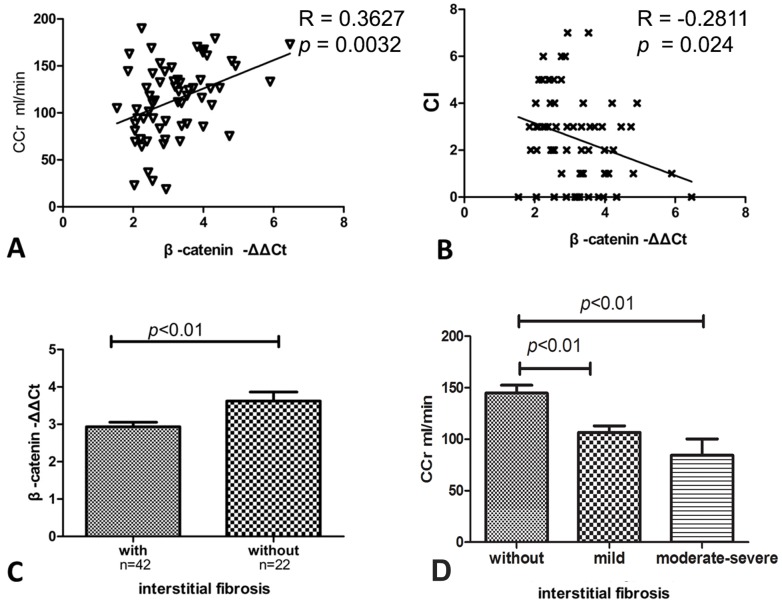
The correlation between gene expression of β-catenin and clinical and laboratory characteristics. The mRNA expression of β-catenin demonstrated a positive correlation with Ccr (*p*<0.01, A) and an inverse correlation with CI (*p*<0.05, B). mRNA expression levels of β-catenin were higher in patients without interstitial fibrosis than in patients with interstitial fibrosis (-ΔΔCt = 3.622±0.240 vs. 2.934±0.124, respectively, *p*<0.01, C). The patients with renal fibrosis had significantly lower Ccr levels than those without (*p*<0.01, D).

We further investigated the relationship of β-catenin mRNA levels with lupus disease activity. No significant correlation was found between gene expression of β-catenin and 24-hour proteinuria, disease activity (SLEDAI) or dosage of corticosteroid. However, gene expression of β-catenin positively correlated with Ccr and negatively correlated with CI, both of which are indicators of the degree of renal injury (Figure 3A, 3B). In addition, mRNA expression of β-catenin was significantly greater in LN patients without renal interstitial fibrosis compared with those with renal interstitial fibrosis (-ΔΔCt = 3.622±0.240 vs. 2.934±0.124, respectively, *p* = 0.006). (Figure 3C). The patients with renal fibrosis had significantly lower Ccr levels than those without (Figure 3D).

### The expression of Dkk-1 in LN patients

**Figure 4:**
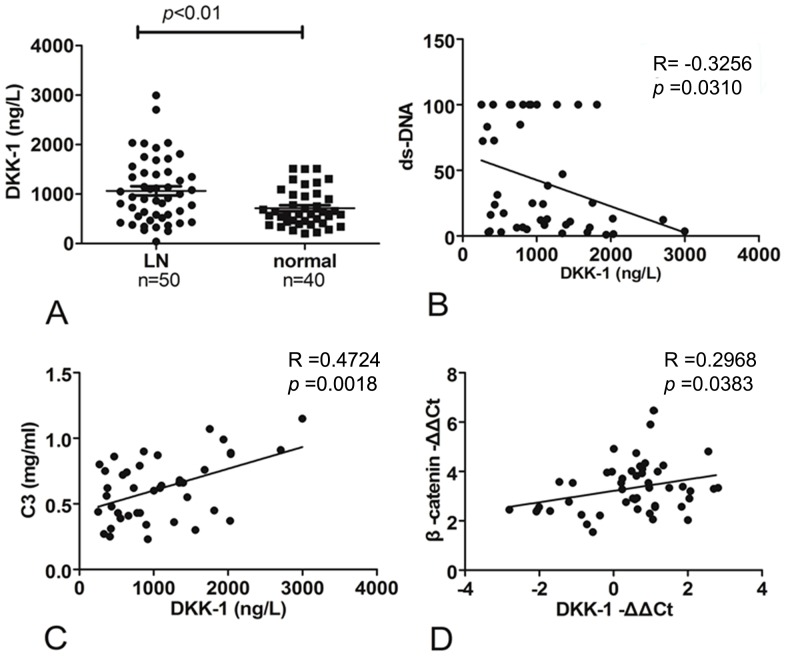
Expression of Dkk-1. Plasma Dkk-1 levels were significantly greater in LN patients compared with those in healthy individuals (1056.3±648.0vs. 714.2±374.9, respectively, *p*<0.01 (A). Plasma Dkk-1 levels negatively correlated with anti-dsDNA antibody concentrations (*p*<0.05, B) and positively correlated with serum C3 concentrations (*p*<0.01, C). mRNA expression of Dkk-1 had a strong positive correlation with β-catenin (*p*<0.05, D).

Dkk-1 is a specific inhibitor of canonical WNT/β-catenin signaling. To further investigate the activity of the WNT pathway during LN, we measured the expression of Dkk-1 in the plasma of 50 of 97 SLE patients and 40 healthy individuals as a control using an ELISA. These results showed that plasma Dkk-1 levels were significantly greater in LN patients compared with normal controls(*p*<0.01 (Figure 4A). Further analysis showed that plasma Dkk-1 concentrations negatively correlated with anti-dsDNA antibody concentrations and positively correlated with serum C3 concentrations (Figure 4B, C).

We also determined mRNA expression levels of Dkk-1, which showed a positive correlation with β-catenin (Figure 4D), although no significant differences were found between LN patients and controls.

## Discussion

Our study showed evidence of increased renal expression of β-catenin by immunohistochemistry and real-time PCR in LN patients, the mRNA expression of Axin2 which was a TCF/LEF-responsive gene can activated byβ-catenin was also increased in LN patients. It was consistent with the study in NZB/NZW mice [7]. This finding suggested that the activation of canonical WNT signaling occurs during LN and that the WNT/β-catenin pathway might play a role in the pathogenesis of LN.

However, the expression of β-catenin did not appear to correlate with clinical parameters, including disease activity, evaluated by SLEDAI, 24-h proteinuria and dosage of corticosteroid. Reasonable interpretations for this finding might be that the SLEDAI assesses multiple organ damage in SLE and that these clinical parameters are quite complex and are affected by a variety of factors, such as duration of disease, current medications and treatment intensity. The interaction between WNT/β-catenin and other pathogenic factors in the pathogenesis of SLE will require further investigation.

β-catenin, the central component of the canonical WNT pathway, forms a transcription activation complex with TCF/LEF that activates the expression of a set of target genes such as c-Myc, TWIST, fibronectin, MMP7, c-Jun, and CTGF; concurrently, the complex represses the expression of E-cadherin and subsequently induces the de novo expression of mesenchymal markers. Cooperation with TGF-β, CTGF, and other signaling pathways results in the downregulation of the epithelial cell-determining gene E-cadherin and the upregulation of the mesenchymal cell-determining genes FSP-1, v-Src, v-ras, c-Fos and other genes, thus promoting the process of EMT. A study by Chunsun Dai [12] showed that hyperactive WNT/β-catenin signaling promoted podocyte dysfunction and proteinuria. All members of the WNT protein family (except WNT5b, WNT8b, and WNT9b), β-catenin, Dkk-1, FZD, fibronectin, and MMP-7 were upregulated in the UUO model of renal fibrosis [13]. Overexpression of Dkk-1 inhibited the activation of β-catenin, fibroblast-specific protein 1 and α-SMA protein, which inhibited the transformation of myofibroblasts and the synthesis of type I collagen, as well as fibronectin, thus reducing collagen deposition [13]. It appeared that blocking the canonical pathway of WNT/β-catenin signaling attenuated renal fibrotic lesions [13].

Ccr and CI are the canonical indicators of renal function, which more severe renal fibrosis is accompanied by lower Ccr and higher CI levels. To verify whether WNT/β-catenin signaling correlated with renal fibrosis, we further divided the LN patients into two groups, according to whether the patients had interstitial fibrosis. Our data showed higher mRNA expression of β-catenin in patients without renal fibrosis than in those with renal fibrosis. Gene expression of β-catenin positively correlated with Ccr and negatively correlated with CI of SLE renal tissue injury. Many reports suggested that WNT/β-catenin signaling promoted fibrotic lesions and lied upstream of factors promoting fibrosis [3], [13].

For the relationship between β-catenin and renal fibrosis,we found higher expression of β-catenin in patients without renal interstitial fibrosis, which was reduced in patients with renal interstitial fibrosis in our study. We also found that patients with renal fibrosis had significantly lower Ccr levels than those without. These data indicated the expression of β-catenin was correlated with the severity of interstitial fibrosis. Benali SL et al. also reported that the number of β-catenin positive tubular epithelial cells was negatively correlated with the severity of chronic interstitial fibrosis grade and serum creatinine concentration in the dog kidney injury model [14]. Their conclusion that β-catenin expression was negatively correlated to fibrosis was in accordance with ours data.

Since non-specific staining was present in some of our samples, which might affect the accuracy of IHC quantification analysis, we applied semiquantitative method to evaluate the expression of β-catenin by an independent pathologist, who was blinded to the clinical data. The mRNA expression level of β-catenin was also found elevated in kidneys from lupus patient as well as the β-catenin protein level of IHC. The mRNA expression of β-catenin was used to analysis with clinical and laboratory parameters. Sometimes, the mRNA level was not always in according with proteins level, it might skew the result. Increasing the sample size of western blotting would reduce the deviation. Dkk-1 is a WNT-responsive gene that was presumed to be a specific negative regulator of WNT signaling [15]. Plasma Dkk-1 levels were greater in LN patients than in controls, which was consistent with the previous study in NZB/NZW mice [7]. Anti-dsDNA antibody is one of

anti-nuclear antibodies and implicated in the pathogenesis of lupus nephritis. Complement component 3(C3) plays a central role in the complement system and contributes to innate immunity. Increased circulating anti-dsDNA antibodies and decreased C3 level predicted higher lupus activity. Our data showed the plasma Dkk-1 concentration inversely correlated with the anti-dsDNA antibody concentration and positively correlated with the serum C3 concentration, indicating that the plasma Dkk-1 concentration was inversely associated with the activity of SLE.

Since Dkk-1 is a downstream of gene of β-catenin, activated β-catenin signaling will lead to upregulation of Dkk-1, which might interpreted the phenomenon of positive correlation between Dkk-1 mRNA expression and expression of β-catenin in LN renal samples. Study on lupus mouse model done also showed a parallel increased levels of Dkk-1 and β-catenin in kidneys [7].

The correlation coefficients were low in our correlation analysis. The possible reason might the heterogeneity of our clinical samples and small sample size. In our future research, increasing sample size and screening patients with more homogenous backgrounds might increase the correlation coefficients.In our study, the increased levels of Dkk-1 in plasma did not correlate with Dkk-1 mRNA expression in the kidneys.β-catenin was widely expressed in the immune system, the resource of plasma Dkk-1 might be produced by multiple systems and affected by many factors expect renal.

### Conclusions

In this study, we found that the expression of β-catenin and Axin2 in the kidneys of patients with lupus nephritis was increased. These findings indicated that the classical WNT/β-catenin pathway might play a role in the pathogenesis of lupus nephritis. β-catenin mRNA expression was higher in patients without renal fibrosis than in those with renal fibrosis; additionally, gene expression of β-catenin significantly correlated with Ccr levels and CI. Dkk-1, an inhibitor of the WNT pathway, was greater in the plasma of LN patients in our study. Considering the relationship between Dkk-1, C3 and anti-dsDNA antibody, our data suggested that Dkk-1 might play a protective role in lupus nephritis. The WNT/β-catenin pathway lies upstream of factors that promote fibrosis, and thus, this pathway might be an important element in the induction of renal fibrosis and, eventually, the progression to end-stage renal disease. Further studies on the WNT/β-catenin pathway will help foster understanding of the pathogenesis of lupus nephritis and aid the search for new therapeutic targets.
